# Uses of Southern Vanilla

**Published:** 1875-10

**Authors:** 


Uses of Southern Vanilla.—Dr. A. W. Miller remarks, in the *American Journal of Pharmacy*, that deer tongue, or Southern vanilla (*Liatris odoratissima*, Willd.) seems destined to become a commercial staple of some importance, chiefly, so far, on account of its large consumption as a flavor for tobacco. It is stated to be also used to some extent in the South for the purpose of preserving clothing, woolen fabrics, etc., from the attacks of moths. He recommends a tincture of the leaves as an agreeable perfume, resembling new-mown hay.

